# Validation of at-the-bedside formulae for estimating ventilator driving pressure during airway pressure release ventilation using computer simulation

**DOI:** 10.1186/s12931-022-01985-z

**Published:** 2022-04-26

**Authors:** Sonal Mistry, Anup Das, Sina Saffaran, Nadir Yehya, Timothy E. Scott, Marc Chikhani, John G. Laffey, Jonathan G. Hardman, Luigi Camporota, Declan G. Bates

**Affiliations:** 1 School of Engineering, University of Warwick, Coventry, CV4 7AL UK; 2 Faculty of Engineering Science, University College London, London, WC1E 6BT UK; 3 Department of Anaesthesiology and Critical Care Medicine, Children’s Hospital of Philadelphia, University of Pennsylvania, Philadelphia, PA USA; 4 Academic Department of Military Anaesthesia and Critical Care, Royal Centre for Defence Medicine, ICT Centre, Birmingham, B15 2SQ UK; 5 Nottingham University Hospitals NHS Trust, Nottingham, NG7 2UH UK; 6 Anaesthesia and Intensive Care Medicine, School of Medicine, NUI Galway, Galway, Ireland; 7 Anaesthesia & Critical Care, Division of Clinical Neuroscience, School of Medicine, University of Nottingham, Nottingham, NG7 2UH UK; 8 Department of Critical Care, Guy’s and St Thomas’ NHS Foundation Trust, London, UK

**Keywords:** Mechanical ventilation, Ventilator-induced lung injury, Airway pressure release ventilation, Driving pressure, Acute respiratory distress syndrome, Computer simulation

## Abstract

**Background:**

Airway pressure release ventilation (APRV) is widely available on mechanical ventilators and has been proposed as an early intervention to prevent lung injury or as a rescue therapy in the management of refractory hypoxemia. Driving pressure ($\Delta P$) has been identified in numerous studies as a key indicator of ventilator-induced-lung-injury that needs to be carefully controlled. $\Delta P$ delivered by the ventilator in APRV is not directly measurable in dynamic conditions, and there is no “gold standard” method for its estimation.

**Methods:**

We used a computational simulator matched to data from 90 patients with acute respiratory distress syndrome (ARDS) to evaluate the accuracy of three “at-the-bedside” methods for estimating ventilator $\Delta P$ during APRV.

**Results:**

Levels of $\Delta P$ delivered by the ventilator in APRV were generally within safe limits, but in some cases exceeded levels specified by protective ventilation strategies. A formula based on estimating the intrinsic positive end expiratory pressure present at the end of the APRV release provided the most accurate estimates of $\Delta P$. A second formula based on assuming that expiratory flow, volume and pressure decay mono-exponentially, and a third method that requires temporarily switching to volume-controlled ventilation, also provided accurate estimates of true $\Delta P$.

**Conclusions:**

Levels of $\Delta P$ delivered by the ventilator during APRV can potentially exceed levels specified by standard protective ventilation strategies, highlighting the need for careful monitoring. Our results show that $\Delta P$ delivered by the ventilator during APRV can be accurately estimated at the bedside using simple formulae that are based on readily available measurements.

**Supplementary Information:**

The online version contains supplementary material available at 10.1186/s12931-022-01985-z.

## Introduction

Airway pressure release ventilation (APRV) is a mode of mechanical ventilation consisting of the application of continuous positive airway pressure with time-cycled pressure releases [1]. APRV is widely available on existing ventilators, and has been proposed as an early intervention to prevent lung injury [2] or as a rescue therapy in the management of refractory hypoxemia [3]. Some benefits of the approach are associated with the fact that it readily permits the preservation of spontaneous breathing by the patient. In a trial of 138 adults with ARDS comparing APRV with standard volume-controlled ventilation (VCV), early APRV improved oxygenation and respiratory system compliance, decreased plateau pressure ($${P}_{plat})$$, and shortened both the duration of ventilation and intensive care unit stay [2]. In the context of COVID-19 ARDS, APRV is being used in some centres as a rescue therapy, and its efficacy is currently being evaluated in a clinical trial (NCT04386369).

While several variants exist, we focus here on APRV as a version of BiLevel ventilation, with extreme inspiratory to expiratory (I:E) inversion and short expiratory times based on expiratory flow characteristics to allow carbon dioxide release without alveolar collapse [2].

While APRV provides higher mean airway pressures, it has been speculated that it might also generate high airway driving pressures $\Delta P$ = (${P}_{plat}$ – ${PEEP}_{tot}$), where ${PEEP}_{tot}$ is the lung pressure at end expiration. $\Delta P$ is a recognised mediator of ventilator induced lung injury that has been associated with mortality in adult ARDS [4, 5]. However, since APRV does not allow full lung deflation, ‘true’ expiratory pressure is not measurable, and the $\Delta P$ delivered by APRV in vivo can never be known exactly in dynamic conditions. While static $\Delta P$ can in theory be measured via an expiratory hold manoeuvre, this can be performed only in paralysed or heavily sedated patients with no spontaneous respiratory activity, an unusual condition in patients undergoing APRV.

Several methods have been suggested to address the above problems. In a recent prospective clinical trial of APRV [2], $$\Delta P$$ was estimated by temporarily switching to volume-controlled ventilation (VCV) and then subtracting the previous monitoring Positive End Expiratory Pressure (PEEP) from measured plateau pressure ($${P}_{plat})$$. Two bedside formulae for estimating $$\Delta P$$ have also been proposed—one based on estimating the contribution of expiratory pressure (analogous to intrinsic PEEP: $${PEEP}_{i}$$) present at the end of the APRV release [6], and another based on the assumption that expiratory flow, volume and pressure decay mono-exponentially [7]. While each of these approaches makes reasonable assumptions, it has to date not been possible to quantify or compare their accuracy.

The aims of this study were to use a high-fidelity computational simulator [8], configured to match data from 90 ARDS patients [8], to establish the value of $\Delta P$ delivered by the ventilator for a number of APRV settings, and to compare these values with the estimates of $\Delta P$ produced by each of the methods described in [2, 6, 7]. This simulator is uniquely well-suited to investigate this question, as it includes 100 independent alveolar compartments with variable pathophysiological characteristics, and allows the $\Delta P$ delivered by the ventilator in APRV to be calculated precisely from the simulated lung pressure waveforms using accepted physiological principles. We note that the $\Delta P$ delivered by the ventilator will be a lower bound on the true $\Delta P$ experienced by the patient in the case of additional spontaneous breathing efforts.

## Methods

### Computational model

**Fig. 1:**
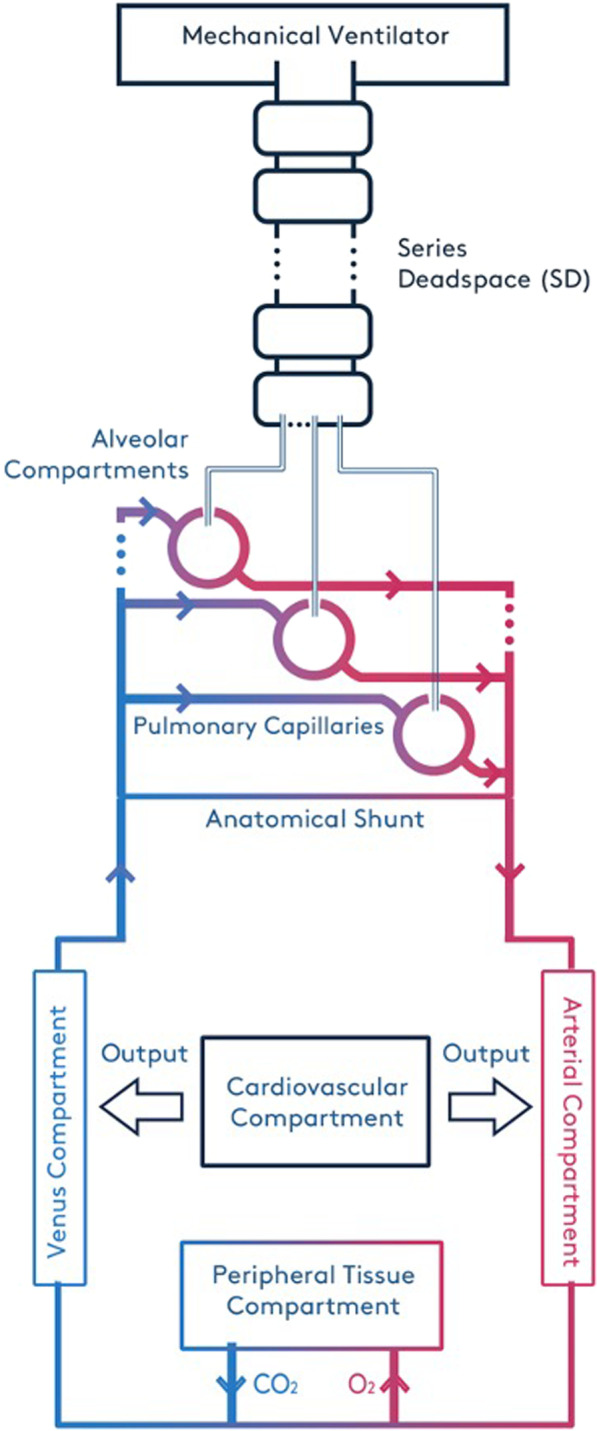
Diagrammatic representation of the model and its main features

The model employed in this investigation has been developed, applied and validated in a number of different studies of ARDS in the past several years [9–16]. This high-fidelity computational simulator is organised into multiple components, each representing different aspects of pulmonary dynamics and blood gas transport—see Fig. 1. These include the transport of air in the mouth, the tidal flow in the airways, gas exchange in multiple alveolar compartments and their corresponding capillary compartments, the flow of blood in the arteries, the veins, the cardiovascular system, and the gas exchange process in the peripheral tissues. Each of these components is comprised of mass conserving functions and iteratively solved as algebraic equations obtained from published literature, experimental data and observational studies. The model includes series dead space (i.e. conducting airways where there is no gas exchange) to represent the trachea, bronchi and the bronchioles, and incorporates 100 independently configurable alveolar compartments, implemented in parallel. Multiple alveolar compartments allow the model to simulate alveolar shunt and alveolar dead space in detail. A full description of the simulator is available in the Additional file 1.

### Patient data

Data were extracted from 90 adult ARDS patients (26% severe, 61% moderate, 13% mild) randomly selected from the low tidal volume arm of the ARMA trial [17]. All patients were fully paralyzed with no spontaneous breathing, received mechanical ventilation in assist-control ventilation mode, and we used the earliest available post-randomization data.

### Model calibration

The model was calibrated against the data on arterial blood gas (ABG) contents, airway pressures and ventilator settings for each individual patient using an optimization approach. The settings of tidal volume (V_T_), fraction of O_2_ in inspiratory air (FiO_2_), and positive end expiratory pressure (PEEP) were fixed at the given values for each patient in the data. Duty Cycle (DC) and respiratory rate (RR), where available, were also set from the data, otherwise, these were also determined through optimization. The model parameters (x) that were used in the optimization include the three key alveolar features (Extrinsic Pressure (*P*_*ext*_), Alveolar Stiffness (*k*_*stiff*_) and Threshold Opening Pressures (*TOP*)) for each of the 100 alveolar compartments, as well as values for respiratory quotient (RQ), total oxygen consumption (VO2), haemoglobin (Hb), volume of anatomical dead space (V_D_) and anatomical shunt (Shunt_anat_). The optimization problem is formulated to find the configuration of model parameters (x) that minimize the difference between the model outputs (for a given set of ventilator settings) and the patient data. This error is captured by a cost function J given below:

1 $$\underset{x}{\mathrm{min}}\nobreakspace J=\sqrt{\sum_{i=1}^{7}\frac{{\widehat{Y}}_{i}-{Y}_{i}}{{Y}_{i}}}$$

where,

2 $$Y = \left[ {PaO_{2} ,PaCO_{2} ,P\prime _{E}CO_{2} ,{PIP},P_{plat},TOP_{mean},V_{frc}} \right].$$

**Fig. 2:**
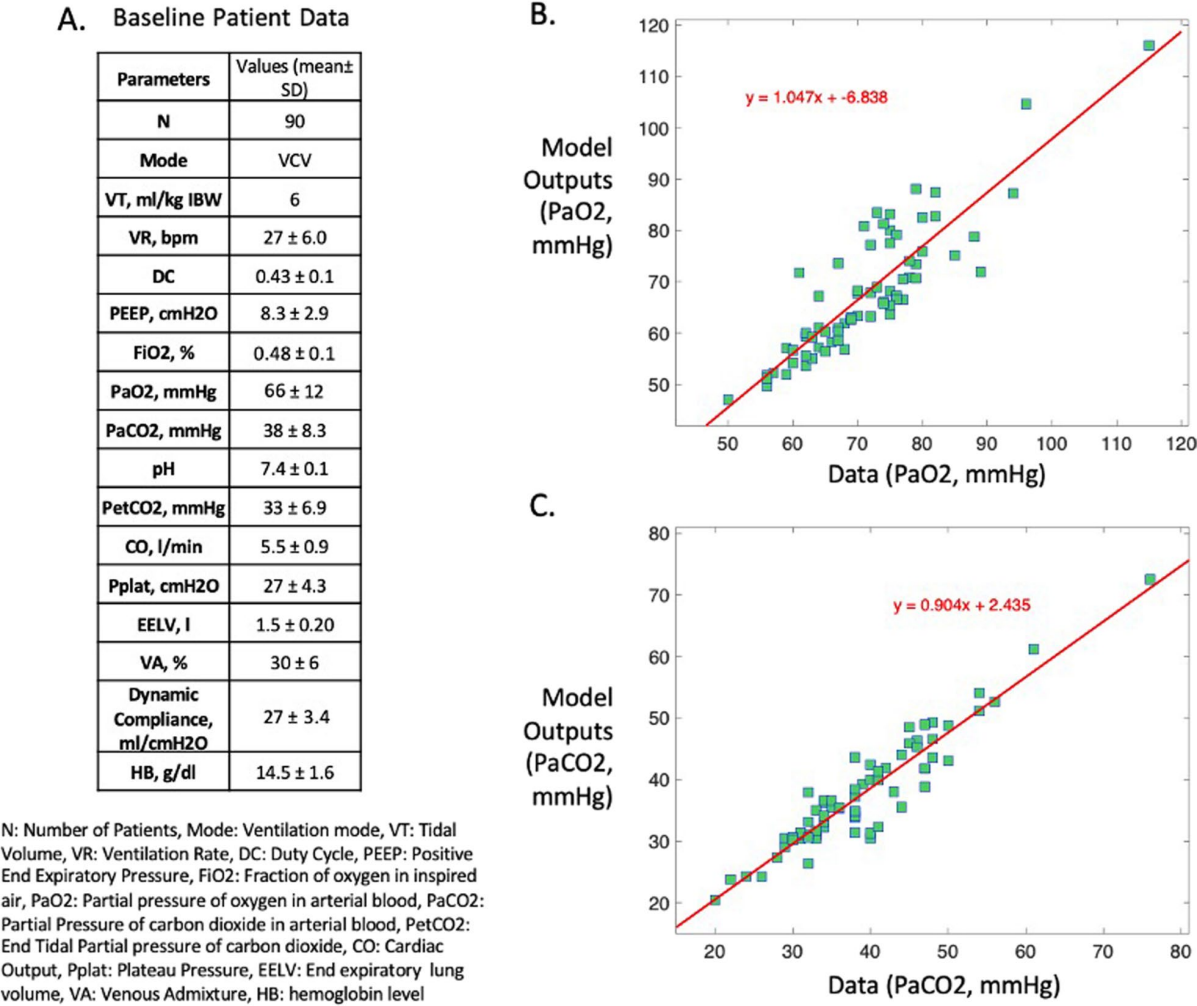
Results of matching the model to the patient data. **A** Baseline patient data for the cohort of 90 ARDS patients, **B** Patient data versus model outputs—PaO_2_, **C** Patient data versus model outputs—PaCO_2_

In the vector of patient data $Y$, PaO_2_ is partial pressure of oxygen, PaCO_2_ is partial pressure of carbon dioxide, Pe’CO_2_ is partial pressure of end-tidal carbon dioxide, PIP is peak inspiratory pressure, TOP_mean_ is average alveolar threshold opening pressure and V_frc_ is functional residual capacity. The vector $\widehat{Y}$ is the corresponding vector of model estimated values. As alveolar threshold opening pressures are not available in the individual patient data, the average threshold opening pressure of all the model compartments (TOP_mean_) is optimized to be 30 cmH_2_O based on data in [18]. Table 1 presents a summary of the parameters included in (x), with their dimensions and allowable ranges of variation. The values of the model parameters (x) that produced the closest match to the patient data were found by using a Genetic Algorithm (GA), a global optimization method. A summary of the results of matching the model to the patient data is shown in Fig. 2. Individual results can be found in Additional file 1: Table S1.

**Table 1:** List of the parameters varied by the optimization algorithm in order to calibrate the model to patient data, with their dimensions and allowable range of variation

Parameter (x)	size	ranges
P_ext_	100	[− 50,28.8]^†^
k_stiff_	100	[− 2,1]^†^
TOP (cmH_2_O)	100	[5,100] [18]
RQ	1	[0.7,0.9] [19]
VO_2_ (mL min^−1^)	1	[150,300] [19]
HB (g.l^−1^)	1	[90,160] [20]
Shunt_anat_ (%)	1	[1, 2] [21]
V_D_ (mL)	1	[60,150] [22]

P_ext_: the extrinsic pressure acting on compartments; k_stiff_: the stiffness of the compartments; TOP: threshold opening pressure of the compartments; RQ: respiratory quotient; VO_2_: total oxygen consumption; HB: hemoglobin; Shunt_anat_: anatomical shunt; V_D_: volume of anatomical dead space

^†^P_ext_ and K_stiff_ ranges were determined to provide a functional residual capacity of 2.5L for an average adult of 70 kg [23]

### Measurements

After matching to the data, the following values were recorded for each simulated patient:

- ${P}_{plat}$ (cmH_2_O), measured from maximum pressure in the lung pressure waveform
- ${P}_{peak}$ (cmH_2_O), measured from maximum pressure in the ventilator pressure waveform
- ${PEEP}_{tot}$ (cmH2O), measured from lung pressure at end expiration in lung pressure waveform
- $${V}_{released}$$ (L), release volume
- ${PEFR}$ (L/min), measured from peak inspiratory flow rate in the flow waveform
- $EEFR$ (L/min), measured from end expiratory flow rate in the flow waveform
- $${V}_{t}$$ (L), tidal volume
- $PF$ ratio (mmHg), calculated as ${PaO}_{2 }/ Fi{O}_{2}$
- ${V}_{frc}$ (ml), calculated as minimum lung volume at end of expiration
- ${R}_{aw}$ (cmH_2_O/L/min), airway resistance calculated as (${P}_{peak}-{P}_{plat}$) / (PeakFlow)
- $C$ (cmH_2_O/L), lung compliance, calculated as (${P}_{plat}-{PEEP}_{tot}$) / (${V}_{t}$)
- $E$ (L/cmH_2_O), lung elastance, calculated as 1/$C$

Each virtual patient was then subjected to APRV ventilation for 30 min, with ${P}_{high}$ and ${P}_{low}$ set to 30 and 5 cmH_2_O, 30 and 0 cmH_2_O, and 25 and 0 cmH_2_O. In accordance with [2], ${T}_{high}$ and ${T}_{low}$ were set to 5 and 0.5 s for the adult cohort. The true $\Delta P$ delivered was calculated as.

3 $$\left( {\Delta P = P_{{plat}} - PEEP_{{tot}} } \right)$$

where ${P}_{plat}$ and ${PEEP}_{tot}$ were measured directly from the lung pressure waveforms described above. ${V}_{released}$, $PEFR,$ and $EEFR$ were also recorded, and estimates of $\Delta P$ during APRV were then calculated as follows:

Under the assumption that full expiration and elimination of ${PEEP}_{i}$ (i.e., achieving zero expiratory flow) requires approximately 4 expiratory time constants ($\tau =resistance \times static \nobreakspace compliance$) [6], ${PEEP}_{i}$ can be estimated as:

4 $${PEEP}_{i}= \left[\frac{1}{2} \left(4\tau \times PEFR\right)-{V}_{ released}\right] \times elastance$$

where $PEFR$ is peak expiratory flow rate, and ${V}_{ released}$ is exhaled volume,

and hence

5 $$\Delta P={P}_{high}-\left({PEEP}_{i}+{P}_{low}\right)$$

We also assessed this method under the assumption that full expiration requires 3 time constants $\tau$.

Alternatively, by making the simplifying assumption that flow, volume and pressure all decay mono-exponentially [7], ${PEEP}_{i}$ and $\Delta P$ can be estimated as:

6 $${PEEP}_{i}= \left(\frac{EEFR}{PEFR}\right)\times {P}_{high}$$

and

7 $$\Delta P= {P}_{high}-\left(\left(\left(\frac{EEFR}{PEFR}\right)\times {P}_{high}\right)+{P}_{low}\right)$$

where $EEFR$ is the flow rate at the end of the pressure release.

To replicate the estimation method used in [2], each simulated patient was switched to VCV for 5 min, with ${V}_{T}$ = ${V}_{released}$ and PEEP = ${PEEP}_{tot}$ used to calculate ${P}_{plat}$ and $\Delta P$ using Eq. (3).

## Results

Across the cohort of 90 patients, true $\Delta P$ delivered was 15.1 ± 2.1 cmH_2_O for APRV settings of ${P}_{high}=30$ cmH_2_O and ${P}_{low}$= 5 cmH_2_O_,_ 17.6 ± 1.4 cmH_2_O for ${P}_{high}=30$ cmH_2_O and ${P}_{low}$= 0 cmH_2_O_,_ and 14.1 ± 1.1 cmH_2_O for ${P}_{high}=25$ cmH_2_O and ${P}_{low}$= 0 cmH_2_O. No correlation across the cohort between levels of true $\Delta P$ delivered and static compliance or PF ratio was observed (Table 2). Mean tidal volumes were 3.11, 3.18 and 3.78 ml/kg for ${P}_{high}$ / ${P}_{low}$= 25 / 0, 30 / 5 and 30 / 0 cmH_2_O, respectively, while mean respiratory rate was 11 breaths/min.

**Table 2:** Pearson correlation coefficients indicating the strength and direction of the relationship between the actual ΔP and baseline lung compliance or baseline PF ratio for different APRV settings

	**Correlation coefficient**
Actual ΔP_adult_ vs Compliance P_high_ = 25 P_low_ = 0	0.00
Actual ΔP_adult_ vs Compliance P_high_ = 30 P_low_ = 0	− 0.11
Actual ΔP_adult_ vs Compliance P_high_ = 30 P_low_ = 5	− 0.07
Actual ΔP_adult_ vs PF ratio P_high_ = 25 P_low_ = 0	− 0.12
Actual ΔP_adult_ vs PF ratio P_high_ = 30 P_low_ = 0	− 0.19
Actual ΔP_adult_ vs PF ratio P_high_ = 30 P_low_ = 5	− 0.07

The correlation coefficients were found to not be statistically significant (*p* < *0.05*)

**Fig. 3:**
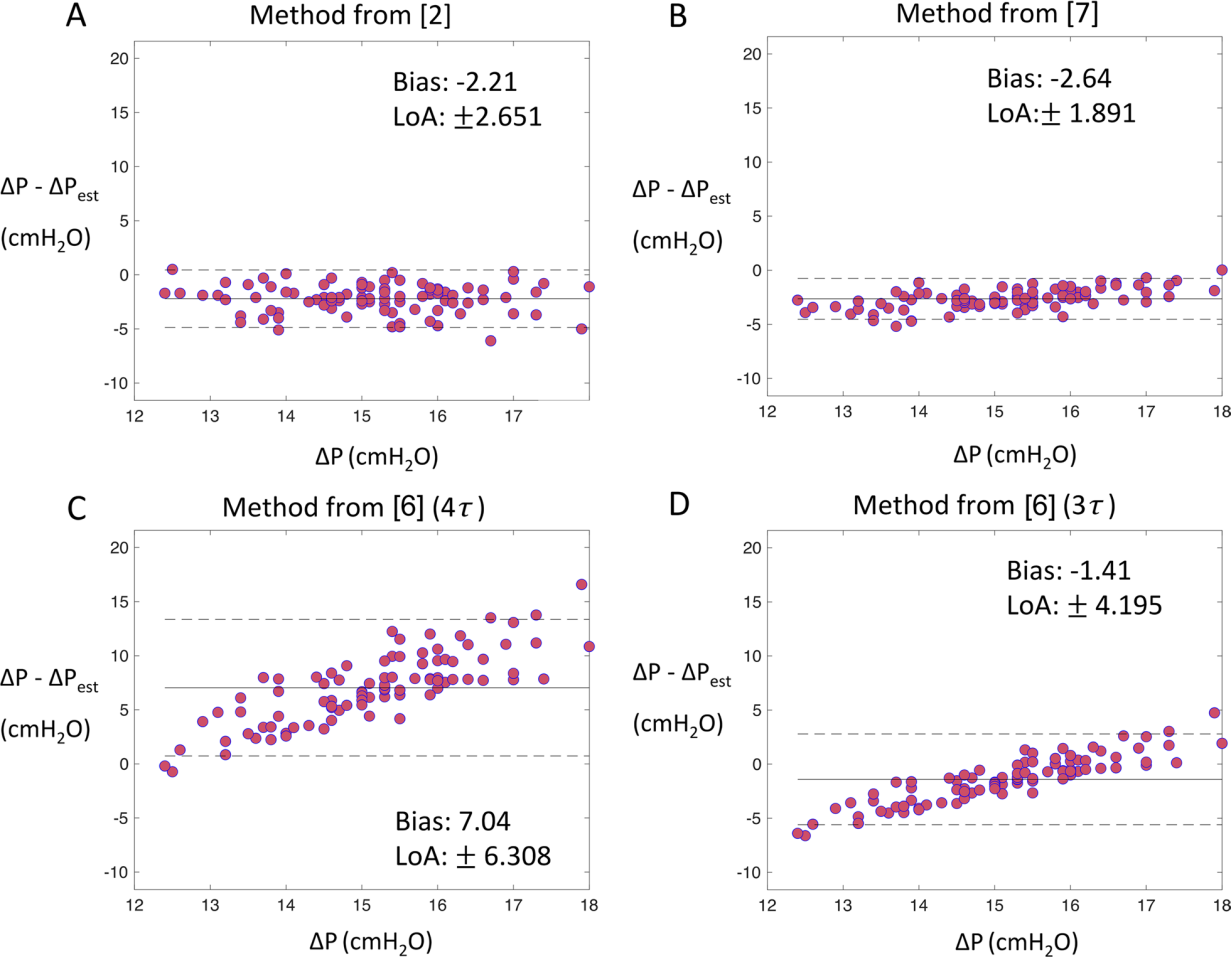
Bland–Altman plots showing estimation error versus true ΔP across the cohort of 90 virtual adult ARDS patients, with ${P}_{high}$= 30 cmH_2_O and ${P}_{low}$ = 5 cmH_2_O. Solid lines indicate the average deviation (‘Bias’) between the true and estimated value of ΔP, dashed lines indicate the limits of agreement (LOA, the distribution of values within ± 1.96 standard deviation from the mean) **A** ΔP estimated by switching to volume controlled ventilation (as described in [2]), assuming that the monitoring PEEP recorded is exactly equal to the actual total expiratory pressure. **B** ΔP estimated using Eqs. (6) and (7), under the assumption that flow, volume and pressure decay mono-exponentially, as proposed in [7]. **C** ΔP estimated using Eqs. (4) and (5) as proposed in [6], under the assumption that the time taken to achieve full expiration and eliminate PEEP_i_ (i.e., achieve zero expiratory flow) is equal to 4 expiratory time constants $\tau .$** D** ΔP estimated as in **C** but under the assumption that the time taken to eliminate PEEP_i_ is equal to 3 expiratory time constants $\tau$

**Fig. 4:**
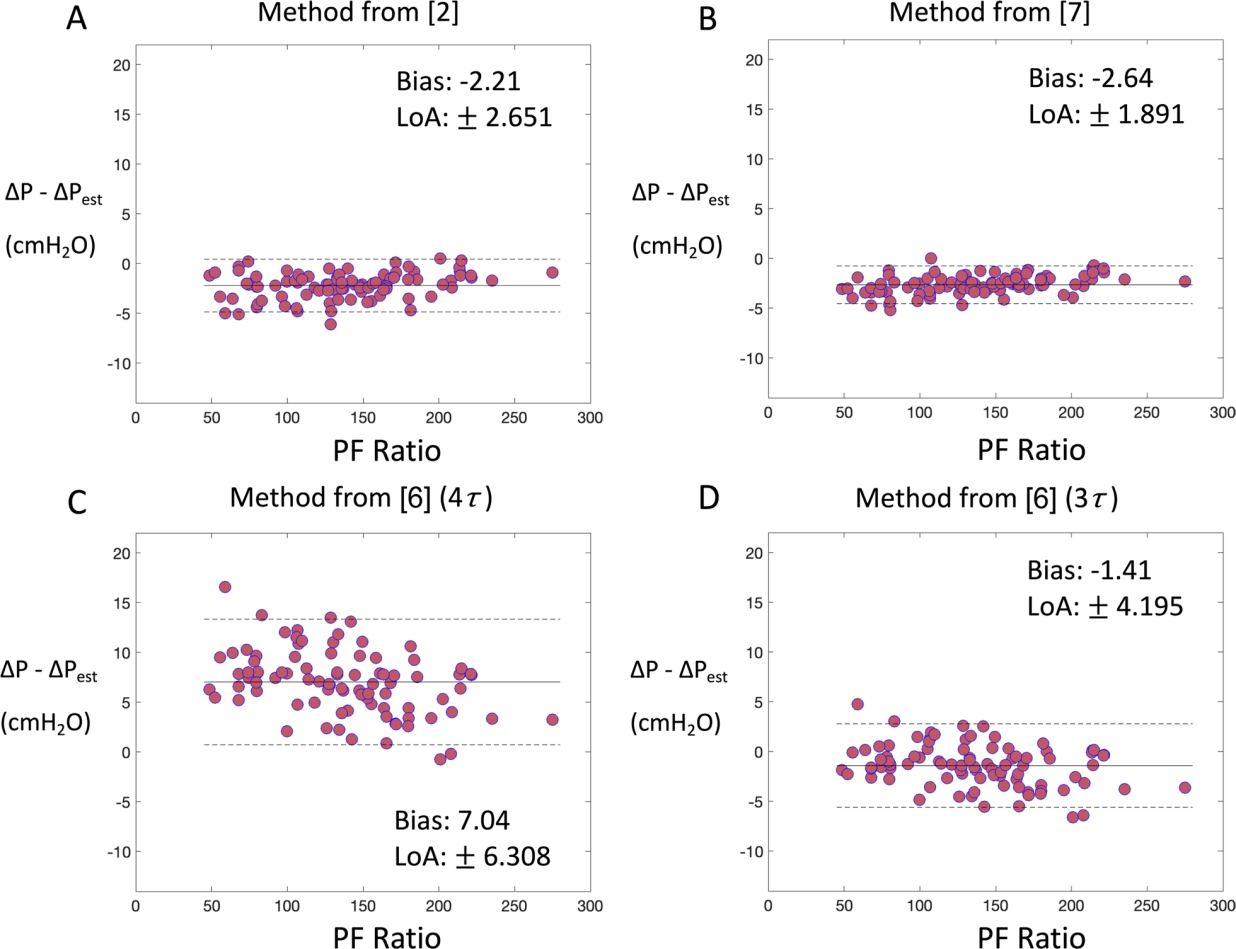
Bland–Altman plots showing estimation error versus PF ratio across the cohort of 90 virtual adult ARDS patients, with ${P}_{high}$= 30 cmH_2_O and ${P}_{low}$ = 5 cmH_2_O. Solid lines indicate the average deviation (‘Bias’) between the true and estimated value of ΔP, dashed lines indicate the limits of agreement (LOA, the distribution of values within ± 1.96 standard deviation from the mean) **A** ΔP estimated by switching to volume controlled ventilation (as described in [2]), assuming that the monitoring PEEP recorded is exactly equal to the actual total expiratory pressure. **B** ΔP estimated using Eqs. (6) and (7), under the assumption that flow, volume and pressure decay mono-exponentially, as proposed in [7]. **C** ΔP estimated using Eqs. (4) and (5) as proposed in [6], under the assumption that the time taken to achieve full expiration and eliminate PEEP_i_ (i.e., achieve zero expiratory flow) is equal to 4 expiratory time constants $\tau .$ **D** ΔP estimated as in (C) but under the assumption that the time taken to eliminate PEEP_i_ is equal to 3 expiratory time constants $\tau$

In each case, the method suggested in [6] gave the least biased estimates of $\Delta P$ on average, but only if Eq. (4) was modified so that zero expiratory flow was achieved after 3, rather than 4, time constants (Fig. 3, Additional file 1: Figures S3 and S4). The approach of [7] produced the tightest limits of agreement (LOA), but consistently overestimated $\Delta P$. The approach of [2] also produced accurate estimates of $\Delta P$, although it requires temporarily switching the patient to VCV, and assumes that the monitoring PEEP equals *PEEP*_*tot*_*.* Accuracy of the estimation methods was unaffected by differences in $PF$ ratio or lung compliance across the cohort (Fig. 4 and Additional file 1: Figures S5–S9).

## Discussion

Our results confirm that $\Delta P$ during APRV can be accurately estimated using simple formulae that only require measurements that are readily available to clinicians at the bedside. The technique proposed in [6] requires the measurement of $PEFR$ (peak expiratory flow rate), ${V}_{ released}$ (exhaled volume), and the lung compliance. ${V}_{ released}$ is measured and available on all ventilators. $PEFR$ is directly measured by some ventilators or can be read from the patient waveforms using the freeze-screen capability of any ventilator. Measurement of lung compliance is routine clinical practice and may be done as described in [6]. The technique proposed in [7] requires only the measurement of $PEFR$ and $EEFR$ (end expiratory flow rate), which may also be calculated using the freeze-screen function of any ventilator. The technique proposed in [2] requires temporarily switching to volume-controlled ventilation (VCV), and then subtracting the previous monitoring PEEP from measured plateau pressure (${P}_{plat}$).

The clinical relevance of $\Delta P$ has not yet been conclusively demonstrated in APRV; rather, its significance is extrapolated from studies using VCV. It is possible that $\Delta P$ during APRV does not have the same prognostic (or mechanistic) significance as it does during conventional ventilation, as the other aspects of ventilation (duration of inspiratory pause, designed air-trapping) are purposefully different in APRV. Nevertheless, given the strong association between $\Delta P$ and mortality in adults on VCV [4, 5], and the difficulty of estimating $\Delta P$ during APRV, it is tempting to speculate whether high $\Delta P$ delivered inadvertently to some patients during previous APRV trials might have contributed to the lack of mortality benefit observed to date.

Our study has some limitations. Our model was calibrated against existing ARDS patient datasets, and we did not perform prospective in vivo assessment of $\Delta P$ during APRV. However, our model has been previously validated [8], and, once calibrated, current state-of-the-art ventilator settings for APRV were applied to evaluate the $\Delta P$ being delivered. The ARDS cohort data and modelling used here assumed no spontaneous breathing, whereas many practitioners prefer to maintain spontaneous ventilation during APRV. The values of $\Delta P$ estimated by the different methods should therefore be considered lower bounds on the true $\Delta P$ being delivered to patients who are also making spontaneous breathing efforts. In the case of additional spontaneous breathing activity, it would be necessary for the clinician to use their judgement as to whether the resulting combined pressures are likely to be injurious—however, this can only be done if some estimate of the $\Delta P$ generated by the ventilator is available. For example, if the estimate of the $\Delta P$ generated by the ventilator is already near the specified limits for protective ventilation, and the patient is also simultaneously making substantial breathing efforts, then this could indicate a potential risk of injurious pressures being delivered. Further clinical studies should be conducted to perform *in-vivo* validation of the estimation methods and clarify the prognostic value of $\Delta P$ during APRV.

## Conclusions

Our results suggest that levels of $\Delta P$ delivered by APRV are generally within safe limits, but that in some cases $\Delta P$ could exceed levels specified by standard protective ventilation strategies, highlighting the need for careful monitoring, particularly in the presence of significant spontaneous breathing efforts by the patient. Our results show that $\Delta P$ delivered by the ventilator during APRV can be accurately estimated at the bedside using simple formulae that are based on readily available measurements.

## Supplementary Information

**Additional file 1.** Detailed model description, additional figures and tables.

## Data Availability

The datasets generated during the current study are available from the corresponding author on reasonable request.

## Abbreviations

APRV: Airway pressure release ventilation
ΔP: Driving pressure
ARDS: Acute respiratory distress syndrome
VCV: Volume controlled ventilation
P_plat_: Plateau pressure
I:E: Inspiratory to expiratory time ratio
PEEP_tot_: Total positive end expiratory pressure
PEEP_i_: Intrinsic positive end expiratory pressure
ABG: Arterial blood gases
V_T_: Tidal volume
FiO_2_: Fraction of inspired oxygen
DC: Duty cycle
RR: Respiratory rate
P_ext_: Extrinsic Pressure
K_stiff_: Alveolar stiffness
TOP: Threshold opening pressure
RQ: Respiratory quotient
TOC: Total oxygen consumption
Hb: Haemoglobin
V_D_: Anatomical dead space
Shunt_anat_: Anatomical shunt
TOP_mean_: Average threshold opening pressure
GA: Genetic algorithm
LOA: Limits of agreement

## References

[1] Lim J, Litton E (2019). Airway pressure release ventilation in adult patients with acute hypoxemic respiratory failure: a systematic review and meta-analysis. Critical Care Med.

[2] Zhou Y (2017). Early application of airway pressure release ventilation may reduce the duration of mechanical ventilation in acute respiratory distress syndrome. Intensive Care Med.

[3] Yener N, Üdürgücü M (2020). Airway pressure release ventilation as a rescue therapy in pediatric acute respiratory distress syndrome. Indian J Pediatr.

[4] Amato MB (2015). Driving pressure and survival in the acute respiratory distress syndrome. N Engl J Med.

[5] Das A, Camporota L, Hardman JG (2019). What links ventilator driving pressure with survival in the acute respiratory distress syndrome? A computational study. Respir Res.

[6] Taylor D, Camporota L (2018). Estimation of true driving pressure during airway pressure release ventilation. Intensive Care Med.

[7] Kenny, J. Driving Pressure in Airway Pressure Release Ventilation: a fool’s errand? https://pulmccm.org/ards-review/driving-pressure-in-airway-pressure-release-ventilation-a-fools-errand/ (PulmCCM, 2018).

[8] Saffaran S (2020). Utility of driving pressure and mechanical power to guide protective ventilator settings in two cohorts of adult and pediatric patients with acute respiratory distress syndrome: a computational investigation. Crit Care Med.

[9] Hardman JG, Aitkenhead AR (1999). Estimation of alveolar deadspace fraction using arterial and end-tidal CO2: a factor analysis using a physiological simulation. Anaesth Intensive Care.

[10] Hardman JG, Bedforth N (1999). Estimating venous admixture using a physiological simulator. Br J Anaesth.

[11] Hardman JG, Bedforth NM, Ahmed AB, Mahajan RP, Aitkenhead AR (1998). A physiology simulator: validation of its respiratory components and its ability to predict the patient's response to changes in mechanical ventilation. Br J Anaesth.

[12] Hardman JG, Aitkenhead AR (2003). Validation of an original mathematical model of CO2 elimination and dead space ventilation. Anesth Analg.

[13] Hardman JG, Wills JS (2006). The development of hypoxaemia during apnoea in children: a computational modelling investigation. Br J Anaesth.

[14] Das A, Gao Z, Menon P, Hardman JG, Bates DG (2010). A systems engineering approach to validation of a pulmonary physiology simulator for clinical applications. J R Soc Interface.

[15] McCahon RA, Columb MO, Mahajan RP, Hardman JG (2008). Validation and application of a high-fidelity, computational model of acute respiratory distress syndrome to the examination of the indices of oxygenation at constant lung-state. Br J Anaesth.

[16] Hardman JG, Al-Otaibi HM (2010). Prediction of arterial oxygen tension: validation of a novel formula. Am J Respir Crit Care Med.

[17] Network TARDS (2000). Ventilation with lower tidal volumes as compared with traditional tidal volumes for acute lung injury and the acute respiratory distress syndrome. N Engl J Med.

[18] Crotti S (2001). Recruitment and derecruitment during acute respiratory failure. Am j Respir Crit Care Med.

[19] Gregory SD, Stevens MC, Fraser JF. *Mechanical circulatory and respiratory support. Ch .1* (Elsevier, London, UK, 2018).

[20] McLellan SA, Walsh TS (2004). Oxygen delivery and haemoglobin. CEACCP.

[21] Dean, L. *Blood Groups and Red Cell Antigens. Ch. 1* (National Center for Biotechnology Information, Bethesda, 2005).

[22] Said SI, Banerjee CM (1963). Venous admixture to the pulmonary circulation in human sublects breathing 100 per cent oxygen. J Clin Invest.

[23] Numa AH, Newth CJ (1985). Anatomic dead space in infants and children. J Appl Physiol.

